# Non-Alcoholic Fatty Liver Disease Is a Risk Factor for the Development of Diabetic Nephropathy in Patients with Type 2 Diabetes Mellitus

**DOI:** 10.1371/journal.pone.0142808

**Published:** 2015-11-13

**Authors:** Guoyu Jia, Fusheng Di, Qipeng Wang, Jinshuang Shao, Lei Gao, Lu Wang, Qiang Li, Nali Li

**Affiliations:** 1 Department of Endocrinology and Metabolism, The Third Central Hospital of Tianjin, Tianjin Key Laboratory of Artificial Cells (TKL), Tianjin, China; 2 Department of Endocrinology and Metabolism, The Third Central Hospital of Tianjin, Tianjin, China; 3 Department of Hemodialysis, The Fourth Central Hospital of Tianjin, Tianjin, China; INRA, FRANCE

## Abstract

**Background:**

Non-alcoholic fatty liver disease (NAFLD) is prevalent in individuals with type 2 diabetes mellitus (T2DM). Diabetic nephropathy (DN) is also associated with T2DM. However, little is known about the interaction between these conditions in patients with T2DM.

**Objective:**

To examine the association between NAFLD and DN in patients with T2DM.

**Methods:**

This retrospective study included patients seen between January 2006 and July 2014.T2DM patients were divided into two groups based on NAFLD status (with NAFLD = group A; without = group B). The cumulative incidence of DN and chronic kidney disease (CKD) staging were compared between the two groups. Liver fat content was examined in some patients. Associations among NAFLD, other factors,and DN were analyzed by the additive interaction method.

**Results:**

Cumulative incidence of DN in patients from group A (58.58%) was higher than in group B (37.22%) (P = 0.005). In both groups, the number of DN patients with CKD stage 1 was greater than the number of patients with stages 2–5. Increased liver fat content was associated with increased occurrence of severe and mild albuminuria and decreased glomerular filtration rate (GFR). There were positive correlations between NAFLD and insulin resistance index (HOMA-IR), free fatty acids (FFA), tumor necrosis factor-α (TNF-α), omentin-1, visceral fat area, homocysteine (HCY), and serum uric acid (UA).

**Conclusion:**

NAFLD might be a risk factor for DN. Elevated liver fat content could be associated with higher DN burden.

## Introduction

Non-alcoholic fatty liver disease (NAFLD) is closely associated with type 2 diabetes mellitus (T2DM).The incidence of NAFLD in the general population is approximately 20–30%, but reaches nearly 75% in patients with T2DM [1]. Diabetic macrovascular complications have been shown to be closely associated with NAFLD [2]. In contrast, little is known about possible associations between NAFLD and diabetic microvascular complications. Diabetic nephropathy (DN) is one of the most common microvascular complications associated with T2DM. However, there is little clinical manifestation at the early stages of the disease. If persistent albuminuria occurs, renal damage is usually irreversible.Therefore, it is critical to monitor individuals with risk factors.Such practice could help identify DN at its early stages, which would likely improve its treatment and outcomes.

Compared with healthy individuals, patients with T2DM are more susceptible to NAFLD. Lu *et al* [3] reported that the incidence of the co-occurrence of T2DM and NAFLD was as high as 75.18% in China. In addition to NAFLD, T2DM is closely associated with a number of metabolic abnormalities such as hypertension, dyslipidemia, and cardiovascular disease [4]. However, most studies about T2DM and NAFLD are cross-sectional studies that failed to demonstrate causality, and most reported conflicting results [5]. One group examined 413 patients with T2DM with or without NAFLD and found no association between NAFLD and the incidence of DN in patients with T2DM [6]. In contrast, two cross-sectional studies conducted by Targher et al [7,8] showed that the incidence of kidney dysfunction was significantly higher in patients with NAFLD.

Given the conflicting findings of various groups, whether or not there is an association between NAFLD and DN is still an open question [9]. Therefore, in an attempt to address some of these questions and limitations, we performed a retrospective cohort study to explore the association between NAFLD and other factors on the development of DN.

## Material and Methods

### Patients

This retrospective cohort study was approved by the Ethics Committee of the Third Central Hospital of Tianjin (Tianjin, China), and all participants provided a written informed consent.Patients with T2DM were retrospectively selected. These patients had been hospitalized several times in the Department of Endocrinology and Metabolism of the Third Central Hospital of Tianjin between January 1, 2006 and July 1, 2014. Medical records of all patients were retrieved and collected according to approved procedures. Patients were divided into two groups: those with T2DM and NAFLD were assigned to group A (n = 169), and those with propensity score-matched T2DM without NAFLD were assigned to group B (n = 169).

Inclusion criteria: Patients were diagnosed with T2DM but without DN at their first admission.The interval between each patient’s first and second admission was seven years. Diagnosis of T2DM was strictly made according to the standards defined by WHO in 1999 [10]. The diagnosis of NAFLD was made according to the2012 statement of the American Gastroenterological Association [11]: 1) hepatic steatosis based on imaging or histology; 2) no significant alcohol consumption; 3) no competing etiologies for hepatic steatosis; and 4) no co-existing causes for chronic liver disease.The diagnosis and clinical staging of DN was based on Mogensen’s staging criteria [12].

Exclusion criteria were: (1) individuals with type 1 diabetes, adult latent autoimmune diabetes, gestational diabetes, or other special types of diabetes; (2) patients with severe infections, fever, or acute or chronic complications associated with diabetes; (3) individuals with NAFLD diagnosis for seven consecutive years; (4) patients who were younger than 30 years of age or older than 45 years of age at the time of their first admission; (5) patients with hypercortisolism (Cushing's syndrome), growth hormone syndrome, or thyroid dysfunction; (6) individuals who had taken diuretics, β blockers, cholesterol-lowering medication, or hormones affecting glucose and lipid metabolism within two weeks before admission; (7) patients who had a severe history of alcohol consumption (ethanol consumption ≥140 g per week for men and ≥70 g per week for women); (8) patients with a history of viral hepatitis, autoimmune hepatitis, alcoholic liver disease, or toxicant-induced liver disease; or (9) individuals with acute or chronic renal disease at the time of their first admission, or those with renal disease secondary to another disease other than diabetes at the time of their second admission.

### Clinical and laboratory examinations

Measurements of height, body weight, waist circumference, hip circumference, and blood pressure were performed for each patient.Body mass index BMI (kg/m^2^) = body weight (kg) / height^2^ (m^2^).Waist-to-hip ratio (WHR) = waist / hip.Visceral fat area was calculated by the biological resistance determination method using a body composition analyzer (Inbody 720, Biospac System Inc., South Korea) [13].

Venous blood was obtained from the antecubital vein, early in the morning after an 8–10 h fast.The blood sample was divided into five tubes containing 3 ml each. Plasma levels of omentin-1, adiponectin, tumor necrosis factor-α (TNF-α), interleukin-6 (IL-6) (determined by ELISA assay, Edibo Beijing Biological Technology Co. Ltd., Beijing, China), and high sensitive C reactive protein (hs-CRP) (determined by immunoturbidimetry assay, CRP ELISA Kit and Immage 800, from Beckman Coulter, Inc., Germany) were measured.A glucose tolerance test (OGTT) was performed after the patient had orally taken 75 g anhydrous glucose. Fasting blood glucose (FBG) and two-hour postprandial blood glucose (2hPG) were measured by the hexokinase method (Audit Diagnostics, Ireland).Glycosylated hemoglobin (HbA1c) was measured by high performance liquid chromatography (automatic analyzer and supporting reagents from ADAMS AIC). Total cholesterol (TC) and triglycerides (TG) were measured by the cholesterol oxidase method and the glycerol-3-phosphate oxidase (GPO) and phenol & aminophenazone (PAP) methods, respectively (Roche Life Science, US).Low-density lipoprotein cholesterol (LDL-C), high-density lipoprotein cholesterol (HDL-C), and very low-density lipoprotein cholesterol (VLDL) were determined by the direct immune suppression method (DESAY diagnostics system Co. Ltd., Shanghai, China). Apolipoprotein A (apoA) and apolipoprotein B (apoB) were measured by immunoturbidimetric method (reagents from Shanghai KELONG bio-engineering Co. Ltd. and DESAY diagnostics system Co. Ltd., Shanghai, China). Free fatty acid (FFA) was measured by enzyme colorimetric method (Sekisui Chemical Co. Ltd., Japan).Serum creatinine (Scr) was measured by an enzymatic method (creatinine plus Kit, Roche Life Science, US).Levels of blood urea nitrogen (BUN) were determined by the urease test (urease UV rate method, Roche Life Science, US).Levels of serum uric acid (UA) (enzymatic method, Roche Life Science, US) and serum homocysteine (HCY) were determined by immunoassay (performance rate method, Osama Pharmaceutical Co. Ltd., Shenzhen, China).

Insulin and C peptide were measured by the direct chemical luminescence method (Siemens, USA).An insulin resistance index was calculated using the homeostasis model assessment of insulin resistance index (HOMA-IR) = FBG × FINS/22.5 [14]. The urinary albumin excretion rate (UAER) was determined by rate nephelometry using 24-hour urine. The glomerular filtration rate (GFR) was estimated by using the simplified MDRD equation: GFR [ml/ (min 1.73 m^2^)] = 186 × (Scr) ^-1.154^ × (age) ^-0.203^× 0.742 (female) [15].

For liver ultrasound image acquisition and fat content quantification,all measurements were obtained by a single experienced sonographer using the same equipment (Philips type HD-11 B ultrasound detector, Royal Dutch Philips Electronics Ltd., The Netherlands). The final value was the average read out of three independent measurements. Fasted patients were placed in supine and lateral positions, and measurements were obtained using constant parameter setups, including ‘gain’, ‘depth’, and ‘time compensation gain’. Sagittal ultrasound images of the liver,the right kidney, and the right hepatic lobe intercostal section were acquired with an ultrasonic probe positioned in the right rib arch at the anterior axillary line; all acquired images were stored.Ultrasound images were processed with the Image J software (Image J 1.48, USA).A liver parenchyma region of 1.5×1.5 cm was selected from the right kidney sagittal diagram.The region of interest from the renal cortex was selected with an area of 0.5×0.5 cm at the equivalent plane to the selected liver region (the region of interest avoided great vessels, bile duct, renal pelvis, renal medulla structure, and the region with abnormal weakened or strengthened ultrasound signals). Next, the average gray intensity value was calculated for the region of interest (ROI) in the liver and kidney, respectively, to estimate the hepatorenal echo ratio (hepatorenal echo ratio = average echo intensity of liver ROI / average echo intensity of renal ROI from the same plane of liver ROI). The intercostal section of the sonographic image of the liver’s right lobe was selected, and a vertical line was made to the tangent of the sonographic fan image. A liver ROI of 1.5×1.5cm was then determined from the proximate region of the trailing edge at the position of a near field depth of 4 to 6 cm and by the same ultrasound beam. The distance between each sampling frame was measured to calculate the liver attenuation coefficient based on the formula: liver attenuation coefficient = (ln average gray intensity of liver from near field measurements -ln average gray intensity of liver from far field measurements) / (distance between sampling frames × probe frequency). The liver fat content was determined using: liver fat content (%) = 62.592 × standard hepatorenal echo ratio + 168.076 × standard liver echo attenuation coefficient - 27.863 [16].

Patients with T2DM and NAFLD were further divided into subgroups based on their liver fat content. Patients in group A1 had liver fat content ranging from 5.95% to 26.84%, those in group A2 had liver fat content ranging from 26.74% to 38.78%, and those in group A3 had liver fat content ranging from 39.04% to 84.38%.

### Statistical analysis

Propensity score matching (PSM) was achieved using the PSM extension of the SPSS software. Propensity score matching method: using the 1:1 nearest neighbor matching method and caliper value of 0.1 to find the matched subjects with group A as the reference group. In total, 169 pairs of patients were successfully identified. The imbalance of age, body mass, BMI, TG, and VLDL between the two groups was processed by PSM and, eventually, an equilibrium was reached (*P*>0.05). PSM was used in the present study to correct the selection bias and eliminate confounding factors [17].

SPSS 17.0 (IBM, USA) was used for statistical analysis. Continuous data were tested for normality and are presented as means ± standard deviation (SD). Comparison between multiple groups was performed by analysis of variance (ANOVA), and intergroup comparison was performed using t tests. Categorical data are presented as percentage and were analyzed by χ^2^ test. Logistic regression analysis was performed to analyze the factors associated with DN. *P*<0.05 was considered to be statistically significant.

## Results

### Baseline information

A total of 465 patients were initially selected, including 176 patients with NAFLD and 289 patients without NAFLD. Of these, 169 matched pairs of patients were finally recruited after PSM processing.Patients with and without NAFLD were classified into groups A and B, respectively. Differences in age, body mass, BMI, TG, and VLDL between the two groups were processed by PSM and, eventually, equilibrium between the two groups was reached (*P*>0.05) (Table 1).

**Table 1:** Baseline datafor group A and group B

Factors	Before matching			After matching		
	Group A	Group B	P	Group A	Group B	P
N	176	289		169	169	-
Gender (M/F)	95/81	162/127	0.662	92/77	94/75	0.827
Disease course (months)	36.47±5.09	36.02±5.01	0.351	36.48±5.27	35.83±4.92	0.242
Age (years)	53.37±9.62	55.93±10.34	0.008*	53.96±10.55	55.19±9.43	2.259
Height (cm)	169.94±10.09	168.82±9.17	0.219	170.48±6.67	169.18±6.65	0.074
Body mass (kg)	74.33±12.04	70.56±11.07	0.001*	74.58±10.45	73.27±10.06	0.241
BMI (kg/m^2^)	25.91±3.02	23.77±3.80	<0.001*	25.79±4.21	25.71±4.06	0.859
Waistline (cm)	88.52±8.02	86.84±10.80	0.074	88.50±6.78	87.21±7.42	0.096
WHR	0.95±0.11	0.92±0.19	0.057	0.94±0.15	0.92±0.13	0.191
SBP (mmHg)	132.97±15.84	132.08±14.25	0.532	130.07±4.29	129.32±4.24	0.242
DBP (mmHg)	69.01±9.34	67.98±9.97	0.269	68.96±9.65	67.65±10.35	0.230
FBG (mmol/L)	7.56±4.19	7.29±3.57	0.459	7.54±2.41	7.30±2.36	0.356
P2hBG (mmol/L)	13.03±6.22	12.67±5.69	0.351	12.82±3.48	12.55±3.10	0.452
HbA1c (%)	7.62±1.42	7.47±0.97	0.177	7.60±0.93	7.51±1.00	0.392
TC (mmol/L)	4.89±0.94	4.93±1.23	0.711	4.88±0.99	4.95±1. 29	0.576
TG (mmol/L)	2.09±0.63	1.93±0.59	0.006	2.08±0.90	2.08±0.93	1
HDL-C (mmol/L)	1.11±0.41	1.13±0.37	0.588	1.10±0.50	1.11±0.41	0.841
LDL-C (mmol/L)	2.97±0. 84	2.87±0.80	0.200	2.95±0.73	2.83±0.69	0.121
VLDL (mmol/L)	1.15±0.29	1.06±0.31	0.002	1.15±0.27	1.11±0.30	0.199
BUN (mmol/L)	5.71±2.47	5.52±2.09	0.376	5.69±2.11	5.33±1.97	0.106
AST	—	—	—	17.6±4.8	18.6±7.1	0.30
ALT	—	—	—	19.0±7.9	25.5±13.4	<0.01
Cr (μmol/L)	61.21±10.87	60.08±11.49	0.294	61.03±11.07	59.60±12.34	0.263
UAER (±g/min)	9.19±3.98	8.99±3.09	0.545	9.22±3.97	8.68±3.65	0.194
GFR (ml/min/1.73m^2^)	100.81±8.21	101.33±9.93	0.638	101.97±8.19	104.73±9.53	0.070
Fibrosis score	—	—	—	0.286	-1.296	<0.01

Group A: T2DM with NAFLD; group B: T2DM without NAFLD; BMI: body mass index; WHR: waist hip ratio; SBP: systolic blood pressure; DBP: diastolic blood pressure; FBG: fasting blood glucose; P2hBG: 2h postprandial blood glucose; HbA1c: glycosylated hemoglobin; TC: total cholesterol; TG: triglyceride; HDL-C: high density lipoprotein cholesterol; LDL-C: low density lipoprotein cholesterol; VLDL: very low density lipoprotein; BUN: urea nitrogen; Cr: creatinine; UAER: urinary albumin excretion rate; GFR: glomerular filtration rate.

* *P*< 0.05.

### Relationship between NAFLD, DN, and CKD staging

**Fig 1:**
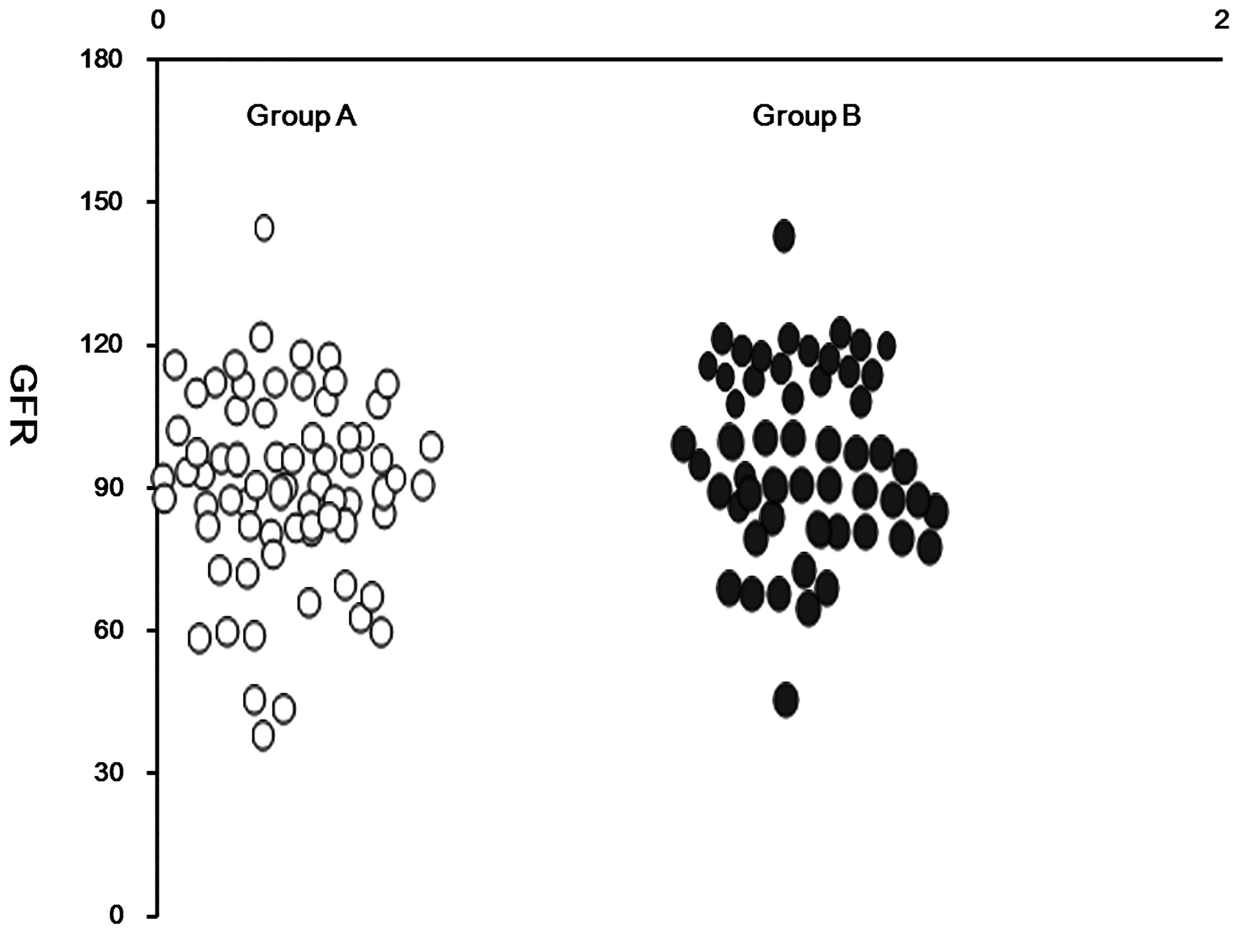
Scatter plot showing the levels of eGFR in the two matched groups.

The cumulative incidence of DN in group A was 58.58%, compared with 39.64% in group B. Therefore, the relative risk (RR) of NAFLD in patients with T2DM was 58.58/39.64 = 1.48.The attributable risk (AR) was 58.58–39.64 = 18.94%.The percentage of attributable risk (ARP) was (58.58–39.64) × 100/58.58 = 32.33% (*P*<0.001; Table 2, Fig 1).

**Table 2:** Relationship between DN,CKD, and NAFLD

	Group A	Group B	P
DN complication	99(58.58%)	67(39.64%)	<0.001*
CKD stage			0.039*
CKD1	65(65.66%)	56(83.58%)	
CKD2	28(28.28%)	9(13.43%)	
CKD3-5	6(6.06%)	2(2.99%)	

* *P*< 0.05.

Fewer patients in group A (65.66%) were CKD1 stage compared to patients in group B (83.58%). In contrast, there was a higher percentage of CKD2 (28.28%) or CKD3-5 (13.43%) in group A compared with group B (6.06% and 2.99%, respectively) (*P* = 0.039; Table 2).

### Relationship between NAFLD severity and renal function

In patients with T2DMandNAFLD, the liver fat content ranged from 5.95% to 84.38%, with a mean of 33.98±15.72%. The cumulative incidence of severe albuminuria and microalbuminuria increased successively in subgroups A1, A2, and A3 (8.93%, 10.71%, and 19.30%, respectively) (*P* = 0.029; Table 3). In contrast, GFR successively decreased in subgroups A1, A2, and A3 (*P*<0.05) (Table 3).

**Table 3:** Relationship between albuminuria, ΔGFR, and different degrees of NAFLD in three sub-groups.

	Group A1 (n = 56)	Group A2 (n = 56)	Group A3 (n = 57)	P
Albuminuria				0.029
Severe	5(8.93%)	6(10.71%)	11(19.30%)	
Mild	21(37.50%)	26(46.43%)	32(56.41%)	
Normal	30(53.57%)	24(42.86%)	14(24.56%)	
ΔGFR	-4.08±0.61	-12.20±1.31^*^	-20.02±2.76^*^ ^#^	<0.001

Δ represents the difference in GFR between readmission measurement and baseline

* compared with group A1, *P*< 0.05

# compared with group A2, *P*< 0.05

### Interaction between NAFLD and other factors and their influence on DN

Several characteristics including waist circumference, WHR, visceral fat area, FINS, HOMA-IR, FFA, TG, VLDL, APOB, TNF-α, IL-6, omentin-1, HCY, and UA were significantly different between groups A and B (*P*<0.05) (Table 4). Additive interaction analysis was performed to explore the potential interactions between NAFLD and the above-mentioned factors in promoting DN development in patients with T2DM. Logistic regression analysis was performed using DN as the dependent variable and the difference value (D-value) of waist circumference, WHR, visceral fat area, FINS, HOMA-IR, FFA, TG, VLDL, APOB, TNF-α, IL-6, omentin-1, HCY, and UA between the two groups as independent variables. This analysis identified a positive correlation between NAFLD and HOMA-IR, FFA, TNF-α, visceral fat area, omentin-1, HCY, and UA (Table 5).

**Table 4:** Differencesin indicators between group A and group B.

Factors	Group A	Group B	*P*
Δ waistline (cm)	1.29±1.00	1.00±1.08	0.011
ΔWHR	0.22±0.19	0.09±0.09	<0.001
Δ visceral fat area (cm^2^)	9.58±6.33	5.53±4.70	<0.001
ΔFINS (mmol/L)	-0.14±0.52	0.06±0.82	0.008
ΔHOMA-IR	0.96±0.30	0.73±0.64	<0.001
ΔFFA (umol/L)	107.81±129.85	56.08±119.78	<0.001
ΔTG (mmol/L)	0.48±0.10	0.26±0.09	<0.001
ΔVLDL (mmol/L)	0.13±0.22	0.06±0.22	0.004
ΔAPOB (g/l)	0.06±0.05	0.04±0.05	<0.001
ΔTNF-α (ng/L)	2.05±1.36	1.04±2.12	<0.001
ΔIL-6 (mg/L)	23.52±7.09	10.57±6.00	<0.001
Δ omentin-1 (μg/L)	-6.39±3.70	-3.11±3.13	<0.001
ΔHCY (μmol/L)	3.29±3.16	1.14±1.22	<0.001
ΔUA (μmol/L)	31.96±18.51	20.34±13.56	<0.001

Δ represents the difference between readmission measurement and baseline; WHR: waist hip ratio; FINS: fasting blood insulin; HOMA-IR: homeostasis model assessment of insulin resistance index; FFA: free fatty acid; TG: triglyceride; VLDL: very low density lipoprotein; APOB: apolipoprotein B; TNF-α: tumor necrosis factor α; IL-6: interleukin 6; HCY: homocysteine; UA: uric acid

**Table 5:** Influence of the interaction of NAFLD with other factors on DN.

Factor	RR	RERI (95%CI)	AP (95%CI)	S (95%CI)
Δ visceral fat area	11.25	2.169 (0.351,7.849)	0.193 (0.151,0.636)	1.269 (1.006,2.340)
ΔHOMA-IR	8.07	4.980 (0.837,9.123)	0.617 (0.382,0.853)	3.384 (1.384,8.277)
ΔFFA	9.28	3.794 (0.745,8.332)	0.409 (0.083,0.734)	1.845 (1.041,3.617)
ΔTNF-α	7.73	3.353 (0.476,7.183)	0.433 (0.104,0.763)	1.991 (1.094,4.132)
ΔUA	4.25	1.695 (0.397,3.788)	0.399 (0.004,0.795)	2.093 (1.727,6.026)
ΔHCY	3.64	2.357 (0.644,4.069)	0.348 (0.245,0.952)	2.421 (1.146,27.705)
Δ omentin-1	6.45	2.399 (0.776,5.574)	0.372 (0.018,0.762)	1.786 (1.079,3.999)

Note: DRERI: interaction of relative excess risk; AP: attributable proportion of interaction; S: interaction index; HOMA-IR: homeostasis model assessment of insulin resistance index; FFA: free fatty acid; TG: triglyceride; TNF-α: tumor necrosis factor α; HCY: homocysteine; UA: uric acid.

## Discussion

We performed a retrospective study to examine the association between NAFLD and DN in patients with T2DM. Results showed that NAFLD might be a risk factor for DN, and that elevated liver fat content could be associated with greater DN damage. Our findings are supported by previous studies showing an association between liver fat accumulation and kidney dysfunction [7,18]. In general, past research has suggested that T2DM, dyslipidemia, and obesity may all contribute to the occurrence of NAFLD. Our findings suggest that NAFLD is one of the most important factors influencing DN development in patients with T2DM. Subgroup analysis based on liver fat content showed that the cumulative rate of albuminuria and microalbuminuria was increased and GFR was decreased with increasing liver fat content; these results are supported by recent data from Targher et al. [19] showing that patients with non-alcoholic steatohepatitis have decreased glomerular filtration rates.Taken together, these data suggest that screening and treatment of NAFLD at the early stages of T2DM would be of clinical significance for the prevention of DN. However, a previous study reported no association between NAFLD and DN [6], but indicated that T2DM duration, waist circumference, and fasting plasma glucose were important risk factors for DN. A number of factors could be responsible for these differences. Indeed, the study by Zhan et al. [6] was cross-sectional and focused mainly on the association between DN and fatty liver, while the present cohort study aimed to show that patients with DM exposed to fatty liver for a certain time were more susceptible to DN. Cohort studies have more power than cross-sectional ones to show the association between risk factors and a disease. Secondly, the previous study had no specific criterion for disease duration. A cross-sectional study without specific criteria for disease course might not be as convincing. The present study suggests that patients with newly diagnosed DM had a higher risk of DN than those with long disease courses. Third, the previous study diagnosed fatty liver using ultrasound, while the present study quantified the extent of fatty liver using ultrasound. Finally, differences in study population and the lack of matching in this previous study might be, at least in part, responsible for the discrepancy between their results and ours.

Disease occurrence is typically influenced by multiple causative factors.Additive interaction analysis is an appropriate method for understanding and evaluating biological interactions. This method revealed that DN results from the interaction of multiple factors [20]. Here, we found positive correlations between NAFLD and HOMA-IR, FFA, TNF-α, visceral fat area, omentin-1, HCY, and UA.Our findings imply that NAFLD combined with any of the above factors would increase the risk of DN in patients with T2DM. Therefore, patients with T2DM and NAFLD should be closely monitored for the above-mentioned factors for better prevention of DN.

Interestingly, we observed that patients with NAFLD showed insulin resistance and increased visceral fat area, which are common components of the metabolic syndrome [21,22]. Recent advances in the field suggest that the metabolic syndrome is an important contributor to the development and progression of chronic kidney dysfunction. Others have reported a positive association between insulin resistance and kidney dysfunction [23,24]. Insulin resistance may also contribute to NAFLD pathogenesis [25].

However, the precise mechanisms by which NAFLD might promote DN are unclear. Additionally, whether NAFLD and DN simply share some common risk factors or whether there is causal relationship between the two is currently unknown. Additional studies must be conducted to address these questions. Along these lines, it should be noted that our study is not without limitation. For example, the sample size was fairly limited. Future studies would benefit from larger multicenter studies.

In conclusion, in this sample of patients with T2DM, NAFLD was one of the most important factors influencing DN development.The diagnosis of NAFLD and the treatment of NAFLD and its interacting factors could be of great clinical significance to help prevent DN in patients with T2DM.

## Supporting Information

S1 Dataset (XLSX)

## References

[1] Leite NC, Salles GF, Araujo AL, Villela-Nogueira CA, Cardoso CR. Prevalence and associated factors of non-alcoholic fatty liver disease in patients with type-2 diabetes mellitus. Liver Int. 2009;29: 113–119. 10.1111/j.1478-3231.2008.01718.x 18384521

[2] Gaggini M, Morelli M, Buzzigoli E, DeFronzo RA, Bugianesi E, Gastaldelli A. Non-alcoholic fatty liver disease (NAFLD) and its connection with insulin resistance, dyslipidemia, atherosclerosis and coronary heart disease. Nutrients. 2013;5: 1544–1560. 10.3390/nu5051544 23666091 PMC3708335

[3] Lu H, Zeng L, Liang B, Shu X, Xie D. High prevalence of coronary heart disease in type 2 diabetic patients with non-alcoholic fatty liver disease. Arch Med Res. 2009;40: 571–575. 10.1016/j.arcmed.2009.07.009 20082871

[4] Takeuchi Y, Ito H, Komatsu Y, Oshikiri K, Antoku S, Abe M, et al Non-alcoholic fatty liver disease is an independent predictor for macroangiopathy in Japanese type 2 diabetic patients: a cross-sectional study. Intern Med. 2012;51: 1667–1675. 22790124 10.2169/internalmedicine.51.7307

[5] Lv WS, Sun RX, Gao YY, Wen JP, Pan RF, Li L, et al Nonalcoholic fatty liver disease and microvascular complications in type 2 diabetes. World J Gastroenterol. 2013;19: 3134–3142. 10.3748/wjg.v19.i20.3134 23716995 PMC3662955

[6] Zhan YT, Zhang C, Li L, Bi CS, Song X, Zhang ST. Non-alcoholic fatty liver disease is not related to the incidence of diabetic nephropathy in Type 2 Diabetes. Int J Mol Sci. 2012;13: 14698–14706. 10.3390/ijms131114698 23203089 PMC3509605

[7] Targher G, Bertolini L, Rodella S, Zoppini G, Lippi G, Day C, et al Non-alcoholic fatty liver disease is independently associated with an increased prevalence of chronic kidney disease and proliferative/laser-treated retinopathy in type 2 diabetic patients. Diabetologia. 2008;51: 444–450. 18058083 10.1007/s00125-007-0897-4

[8] Targher G, Bertolini L, Chonchol M, Rodella S, Zoppini G, Lippi G, et al Non-alcoholic fatty liver disease is independently associated with an increased prevalence of chronic kidney disease and retinopathy in type 1 diabetic patients. Diabetologia. 2010;53: 1341–1348. 10.1007/s00125-010-1720-1 20369224

[9] Orlic L, Mikolasevic I, Bagic Z, Racki S, Stimac D, Milic S. Chronic kidney disease and nonalcoholic Fatty liver disease-is there a link? Gastroenterol Res Pract. 2014;2014: 847539 10.1155/2014/847539 24729784 PMC3963366

[10] (1999) Definition, diagnosis and classification of diabetes mellitus and its complications Part 1: Diagnosis and classification of diabetes mellitus. Geneva: World Health Organization.

[11] Chalasani N, Younossi Z, Lavine JE, Diehl AM, Brunt EM, Cusi K, et al The diagnosis and management of non-alcoholic fatty liver disease: practice guideline by the American Gastroenterological Association, American Association for the Study of Liver Diseases, and American College of Gastroenterology. Gastroenterology 2012;142: 1592–1609. 10.1053/j.gastro.2012.04.001 22656328

[12] Mogensen CE. Microalbuminuria, blood pressure and diabetic renal disease: origin and development of ideas. Diabetologia. 1999;42: 263–285. 10096778 10.1007/s001250051151

[13] Nordstrand N, Gjevestad E, Dinh KN, Hofso D, Roislien J, Saltvedt E, et al The relationship between various measures of obesity and arterial stiffness in morbidly obese patients. BMC Cardiovasc Disord. 2011;11: 7 10.1186/1471-2261-11-7 21284837 PMC3042421

[14] Bonora E, Targher G, Alberiche M, Bonadonna RC, Saggiani F, Zenere MB, et al Homeostasis model assessment closely mirrors the glucose clamp technique in the assessment of insulin sensitivity: studies in subjects with various degrees of glucose tolerance and insulin sensitivity. Diabetes Care. 2000;23: 57–63. 10857969 10.2337/diacare.23.1.57

[15] Levey AS, Coresh J, Greene T, Stevens LA, Zhang YL, Hendriksen S, et al Using standardized serum creatinine values in the modification of diet in renal disease study equation for estimating glomerular filtration rate. Ann Intern Med. 2006;145: 247–254. 16908915 10.7326/0003-4819-145-4-200608150-00004

[16] Xia MF, Yan HM, He WY, Li XM, Li CL, Yao XZ, et al Standardized ultrasound hepatic/renal ratio and hepatic attenuation rate to quantify liver fat content: an improvement method. Obesity (Silver Spring). 2012;20: 444–452. 22016092 10.1038/oby.2011.302 PMC3270296

[17] Thoemmes FJ, Kim ES. A systematic review of propensity score methods in the social sciences. Multivariate Behavioral Research. 2011;46: 90–118. 26771582 10.1080/00273171.2011.540475

[18] Mikolasevic I, Racki S, Bubic I, Jelic I, Stimac D, Orlic L. Chronic kidney disease and nonalcoholic Fatty liver disease proven by transient elastography. Kidney Blood Press Res. 2013;37: 305–310. 10.1159/000350158 24029696

[19] Targher G, Bertolini L, Rodella S, Lippi G, Zoppini G, Chonchol M. Relationship between kidney function and liver histology in subjects with nonalcoholic steatohepatitis. Clin J Am Soc Nephrol. 2010;5: 2166–2171. 10.2215/CJN.05050610 20724519 PMC2994076

[20] Furukawa M, Gohda T, Tanimoto M, Tomino Y. Pathogenesis and novel treatment from the mouse model of type 2 diabetic nephropathy. ScientificWorldJournal. 2013;2013: 928197 10.1155/2013/928197 23737732 PMC3655660

[21] Kim CH, Younossi ZM. Nonalcoholic fatty liver disease: a manifestation of the metabolic syndrome. Cleve Clin J Med. 2008;75: 721–728. 18939388 10.3949/ccjm.75.10.721

[22] Starley BQ, Calcagno CJ, Harrison SA. Nonalcoholic fatty liver disease and hepatocellular carcinoma: a weighty connection. Hepatology. 2010;51: 1820–1832. 10.1002/hep.23594 20432259

[23] Chen J, Muntner P, Hamm LL, Fonseca V, Batuman V, Whelton PK, et al Insulin resistance and risk of chronic kidney disease in nondiabetic US adults. J Am Soc Nephrol. 2003;14: 469–477. 12538749 10.1097/01.asn.0000046029.53933.09

[24] Chen J, Muntner P, Hamm LL, Jones DW, Batuman V, Fonseca V, et al The metabolic syndrome and chronic kidney disease in U.S. adults. Ann Intern Med. 2004;140: 167–174. 14757614 10.7326/0003-4819-140-3-200402030-00007

[25] Milic S, Stimac D. Nonalcoholic fatty liver disease/steatohepatitis: epidemiology, pathogenesis, clinical presentation and treatment. Dig Dis. 2012;30: 158–162. 10.1159/000336669 22722431

